# Large-scale circulating proteome association study (CPAS) meta-analysis identifies circulating proteins and pathways predicting incident hip fractures

**DOI:** 10.1093/jbmr/zjad011

**Published:** 2024-01-04

**Authors:** Thomas R Austin, Howard A Fink, Diana I Jalal, Anna E Törnqvist, Petra Buzkova, Joshua I Barzilay, Tianyuan Lu, Laura Carbone, Maiken E Gabrielsen, Louise Grahnemo, Kristian Hveem, Christian Jonasson, Jorge R Kizer, Arnulf Langhammer, Kenneth J Mukamal, Robert E Gerszten, Maria Nethander, Bruce M Psaty, John A Robbins, Yan V Sun, Anne Heidi Skogholt, Bjørn Olav Åsvold, Rodrigo J Valderrabano, Jie Zheng, J Brent Richards, Eivind Coward, Claes Ohlsson

**Affiliations:** Cardiovascular Health Research Unit, University of Washington, Seattle, WA, 98195, United States; Geriatric Research Education and Clinical Center, VA Health Care System, Minneapolis, MN, 56401, United States; Division of Nephrology, Department of Internal Medicine, Carver College of Medicine, Iowa City, IA, 52242, United States; Iowa City VA Medical Center, Iowa City, IA, 52246, United States; Department of Internal Medicine and Clinical Nutrition, Institute of Medicine, Sahlgrenska Osteoporosis Centre, Centre for Bone and Arthritis Research at the Sahlgrenska Academy, University of Gothenburg, 413 45, Gothenburg, Sweden; Department of Biostatistics, University of Washington, Seattle, WA, 98115, United States; Division of Endocrinology, Kaiser Permanente of Georgia, Atlanta, GA, 30339, United States; Lady Davis Institute for Medical Research, Jewish General Hospital, Montreal, Quebec, H3T 1E2, Canada; Quantitative Life Sciences Program, McGill University, Montreal, Quebec, H3G 0B1, Canada; 5 Prime Sciences Inc, Montreal, Quebec, H3Y 2W4, Canada; Charlie Norwood VAMC, Augusta, GA, 30901, United States; Department of Medicine, Medical College of Georgia, Augusta University, Augusta, GA, 30912, United States; Department of Public Health and Nursing, K.G. Jebsen Center for Genetic Epidemiology, Norwegian University of Science and Technology, 7491, Trondheim, Norway; Department of Internal Medicine and Clinical Nutrition, Institute of Medicine, Sahlgrenska Osteoporosis Centre, Centre for Bone and Arthritis Research at the Sahlgrenska Academy, University of Gothenburg, 413 45, Gothenburg, Sweden; Department of Public Health and Nursing, K.G. Jebsen Center for Genetic Epidemiology, Norwegian University of Science and Technology, 7491, Trondheim, Norway; HUNT Research Centre, NTNU, 7600, Levanger, Norway; Department of Public Health and Nursing, K.G. Jebsen Center for Genetic Epidemiology, Norwegian University of Science and Technology, 7491, Trondheim, Norway; Cardiology Section, San Francisco VA Health Care System, San Francisco, CA, 94121, United States; Department of Medicine, Epidemiology and Biostatistics, University of California San Francisco, San Francisco, CA, 94158, United States; HUNT Research Centre, NTNU, 7600, Levanger, Norway; Levanger Hospital, Nord-Trøndelag Hospital Trust, 7600, Levanger, Norway; Department of Medicine, Beth Israel Deaconess Medical Center, Brookline, MA, 2446, United States; Department of Medicine, Beth Israel Deaconess Medical Center, Brookline, MA, 2446, United States; Department of Internal Medicine and Clinical Nutrition, Institute of Medicine, Sahlgrenska Osteoporosis Centre, Centre for Bone and Arthritis Research at the Sahlgrenska Academy, University of Gothenburg, 413 45, Gothenburg, Sweden; Bioinformatics and Data Center, Sahlgrenska Academy, University of Gothenburg, 413 90, Gothenburg, Sweden; Cardiovascular Health Research Unit, University of Washington, Seattle, WA, 98195, United States; Departments of Medicine, Epidemiology, and Health Systems and Population Health, University of Washington, Seattle, WA, 98195, United States; Department of Medicine, University of California, Davis, CA, 95817, United States; Department of Epidemiology, Rollins School of Public Health, Emory University, Atlanta, GA, 30322, United States; Department of Public Health and Nursing, K.G. Jebsen Center for Genetic Epidemiology, Norwegian University of Science and Technology, 7491, Trondheim, Norway; Department of Public Health and Nursing, K.G. Jebsen Center for Genetic Epidemiology, Norwegian University of Science and Technology, 7491, Trondheim, Norway; Department of Endocrinology, Clinic of Medicine, St. Olavs Hospital, Trondheim University Hospital, 7491, Trondheim, Norway; Research Program in Men’s Health, Aging and Metabolism, Harvard Medical School, Brigham and Women’s Hospital, Boston, MA, 2130, United States; Department of Endocrine and Metabolic Diseases, Shanghai Institute of Endocrine and Metabolic Diseases, Shanghai Jiao Tong University School of Medicine, Ruijin Hospital, Shanghai, 200025, China; Key Laboratory for Endocrine and Metabolic Diseases of the National Health Commission of the PR China, Shanghai Key Laboratory for Endocrine Tumor, Shanghai National Clinical Research Center for Metabolic Diseases, Shanghai Digital Medicine Innovation Center, Shanghai Jiao Tong University School of Medicine, Ruijin Hospital, Shanghai, 200025, China; MRC Integrative Epidemiology Unit (IEU), Bristol Medical School, University of Bristol, Oakfield House, Bristol, BS8 2BN, United Kingdom; Lady Davis Institute for Medical Research, Jewish General Hospital, Montreal, Quebec, H3T 1E2, Canada; 5 Prime Sciences Inc, Montreal, Quebec, H3Y 2W4, Canada; Department of Human Genetics, McGill University, Montreal, Quebec, Canada; Department of Epidemiology, Biostatistics, and Occupational Health, McGill University, Montreal, Quebec, Canada; Department of Medicine, McGill University, Montreal, Quebec, H4A 3J1, Canada; Department of Twin Research, King’s College London, London, SE1 7EH, United Kingdom; Department of Public Health and Nursing, K.G. Jebsen Center for Genetic Epidemiology, Norwegian University of Science and Technology, 7491, Trondheim, Norway; Department of Internal Medicine and Clinical Nutrition, Institute of Medicine, Sahlgrenska Osteoporosis Centre, Centre for Bone and Arthritis Research at the Sahlgrenska Academy, University of Gothenburg, 413 45, Gothenburg, Sweden; Department of Drug Treatment, Region Västra Götaland, Sahlgrenska University Hospital, 413 45, Gothenburg, Sweden

**Keywords:** proteomics, hip fracture, meta-analysis, osteoporosis, LXR/RXR

## Abstract

Hip fractures are associated with significant disability, high cost, and mortality. However, the exact biological mechanisms underlying susceptibility to hip fractures remain incompletely understood. In an exploratory search of the underlying biology as reflected through the circulating proteome, we performed a comprehensive Circulating Proteome Association Study (CPAS) meta-analysis for incident hip fractures. Analyses included 6430 subjects from two prospective cohort studies (Cardiovascular Health Study and Trøndelag Health Study) with circulating proteomics data (aptamer-based 5 K SomaScan version 4.0 assay; 4979 aptamers). Associations between circulating protein levels and incident hip fractures were estimated for each cohort using age and sex-adjusted Cox regression models. Participants experienced 643 incident hip fractures. Compared with the individual studies, inverse-variance weighted meta-analyses yielded more statistically significant associations, identifying 23 aptamers associated with incident hip fractures (conservative Bonferroni correction 0.05/4979, *P* < 1.0 × 10^−5^). The aptamers most strongly associated with hip fracture risk corresponded to two proteins of the growth hormone/insulin growth factor system (GHR and IGFBP2), as well as GDF15 and EGFR. High levels of several inflammation-related proteins (CD14, CXCL12, MMP12, ITIH3) were also associated with increased hip fracture risk. Ingenuity pathway analysis identified reduced LXR/RXR activation and increased acute phase response signaling to be overrepresented among those proteins associated with increased hip fracture risk. These analyses identified several circulating proteins and pathways consistently associated with incident hip fractures. These findings underscore the usefulness of the meta-analytic approach for comprehensive CPAS in a similar manner as has previously been observed for large-scale human genetic studies. Future studies should investigate the underlying biology of these potential novel drug targets.

## Lay Summary

Hip fractures are associated with significant disability, high cost, and mortality. However, the exact biological mechanisms underlying susceptibility to hip fractures remain incompletely understood. To increase the understanding of the underlying mechanisms, we performed a meta-analysis of the associations between 4860 circulating proteins and risk of fractures using two large cohorts, including 6430 participants with 643 incident hip fractures. We identified 23 proteins/aptamers associated with incident hip fractures. Two proteins of the growth hormone/insulin growth factor system (GHR and IGFBP2), as well as GDF15 and EGFR were most strongly associated with hip fracture risk. High levels of several inflammation-related proteins were also associated with increased hip fracture risk. Pathway analysis identified reduced LXR/RXR activation and increased acute phase response signaling to be overrepresented among those proteins associated with increased hip fracture risk. Future mechanistic studies should investigate the underlying biology of these novel protein biomarkers which may be potential drug targets.

## Introduction

Osteoporosis is one of the most common conditions among the elderly. It is characterized by low bone mass and micro-architectural deterioration of bone tissue, leading to an increased risk of fragility fractures.^1^ Fracture risk is not only determined by bone strength but also by the risk of falls, which is influenced by factors such as muscle mass and balance.^2^^,^^3^ Of all fracture types, hip fractures are associated with the highest mortality risk and also with significant disability.^4^ The incidence of hip fractures increases substantially with age, and given the increasing age of the population, the financial and societal burden will likely rise in the coming years.^5^ However, the exact biological mechanisms underlying susceptibility to hip fractures remain incompletely understood.

Although large-scale genome-wide association studies (GWASs) meta-analyses have successfully identified multiple signals for BMD-related parameters, only five genetic signals for hip fractures have been identified.^6-19^ Proteomics is an alternative method to identify the biology underlying susceptibility to hip fractures. Recent advances in proteomic techniques have enabled quantitative analyses of >5000 proteins using relatively small sample volumes.^20^^,^^21^ Proteins regulate many biological processes, and the circulating levels of proteins integrate the effects of genes with effects caused by the environment, age, comorbidities, behaviors, and drugs.^22^ Since the circulating protein profile is dynamic during life, whereas genes remain the same, it is possible that large-scale circulating proteomics may identify novel biomarkers not previously identified by genetic approaches or signaling pathways.^23^ Up to 2200 proteins enter the bloodstream via secretion to regulate biological processes in health or disease, including hormones, cytokines, chemokines, adipokines, and growth factors. Other proteins enter plasma through cleavage of extracellular domains of membrane proteins or leakage from cell damage and cell death. Secreted, cleaved, and leaked proteins provide information about health status and disease risk.^24^ Proteins represent viable therapeutic targets as approximately 96% of all currently approved medications target proteins.^21^

The aim of the present study was to identify novel circulating protein biomarkers for incident hip fractures that may be used to enhance the understanding of the biological mechanisms underlying the susceptibility to hip fractures. We hypothesized that meta-analyses of Circulating Proteome Association Studies (CPAS meta-analyses) may be useful for comprehensive proteomic studies in a similar manner as has been successfully used in human genetic studies (GWAS meta-analyses).^25^

To identify novel protein biomarkers for hip fractures, we included 6430 participants with 643 incident hip fractures from two prospective cohort studies (the Cardiovascular Health Study [CHS] and the Trøndelag Health Study [HUNT]). Both cohorts used the aptamer-based 5 K SomaScan version 4.0 assay for circulating proteomic analyses, which included as many as 4979 aptamers corresponding to 4860 proteins. The CPAS results from the two cohorts were combined using inverse-variance weighted (IVW) meta-analysis.

## Materials and methods

### Cohorts

We evaluated 6430 individuals (643 incident hip fracture cases) with data on circulating proteins from two prospective cohort studies: CHS (mean age 74.4 yr, 60.8% women^26^) and HUNT (mean age 64.5 yr 39.4% women^27^^,^^28^) (Table 1).

**Table 1:** Cohort summary statistics.

	CHS						HUNT						ALL subjects					
	Combined		Men		Women		Combined		Men		Women		Combined		Men		Women	
*n*	3171		1244		1927		3259		1975		1284		6430		3219		3211	
Age [yr]	74.4	(4.9)	74.8	(5.1)	74.1	(4.7)	64.5	(10.1)	63.7	(10.0)	65.8	(10.0)	69.4	(9.4)	68.0	(10.0)	70.8	(8.4)
Height [cm]^a^	164.2	(9.5)	173.0	(6.6)	158.6	(6.2)	170.8	(9.3)	176.2	(6.6)	162.4	(6.0)	167.5	(10.0)	175.0	(6.8)	160.1	(6.4)
Weight [kg]^a^	72.1	(14.3)	79.2	(12.3)	67.4	(13.6)	81.7	(14.9)	87.2	(13.1)	73.2	(13.4)	77.0	(15.4)	84.1	(13.4)	69.7	(13.8)
BMI [kg/m^2^]^a^	26.7	(4.5)	26.5	(3.7)	26.8	(5.0)	27.9	(4.2)	28.0	(3.7)	27.7	(4.8)	27.3	(4.4)	27.4	(3.8)	27.2	(4.9)
Follow-up time [yr]	12.6	(6.3)	11.7	(6.2)	13.2	(6.3)	11.5	(3.6)	11.5	(3.6)	11.5	(3.5)	12.1	(5.1)	11.6	(4.8)	12.5	(5.4)
Incident hip fractures [*n*]	456		133		323		187		76		111		643		209		434	

Mean values are given with SD in parentheses.

a Height and/or weight measurements are missing for 19 HUNT participants and 18 CHS participants.

CHS, Cardiovascular Health Study; HUNT, Trøndelag Health Study.

### The Cardiovascular Health Study

CHS is a population-based longitudinal study of heart disease and stroke in adults aged 65 yr and older recruited from four US communities: Forsyth County, North Carolina; Sacramento County, California; Washington County, Maryland; and Pittsburgh, Pennsylvania.^26^ At baseline (1989-1990), 5201 individuals were enrolled. An additional 687 African Americans were recruited during 1992-1993. Clinic examinations were performed at study baseline and at annual visits through 1998-1999, and again in 2005-2006. Participants were contacted by telephone annually mid-way between exams, and twice per year during 2000-2004 when no clinic examinations occurred. CHS participants are still being contacted twice yearly. EDTA-plasma was collected from fasting blood samples in 1992-1993 and stored at −70 to −80 °C until proteomic profiling of previously unthawed EDTA-plasma was performed. Of 5888 participants enrolled in the study, 5265 were present for the 1992-1993 exam. In 2020, proteomic profiling was performed in 3171 of those participants using a SomaScan panel (5 K SomaScan version 4.0 assay).^29^ Each participant gave informed consent, and each center underwent institutional review board approval.

### The Trøndelag Health Study

HUNT comprises data and samples obtained through four population studies between 1984 and 2019.^27^^,^^28^ About 230 000 people from the Norwegian county of Trøndelag completed self-reported questionnaires, and almost 120 000 participants submitted biological samples. More than 120 000 completed anthropometric measurements such as height, weight, and blood pressure. For proteomic analyses, non-fasting plasma samples were collected at the third HUNT visit (HUNT3; 2006-2008) and stored at −80 °C until proteomic profiling of previously unthawed EDTA-plasma was performed in 2017, using the same SomaScan panel as CHS. Proteomic profiling was performed in 3259 participants from a HUNT cardiovascular project, including 1270 participants with and 1989 participants without incident cardiovascular events. Each participant gave informed consent, and the present study was approved by the Regional Committee for Medical and Health Research Ethics (REK Central Norway 2015/615).

### Proteomics

The 5 K SomaScan version 4.0 aptamer-based assay was used to measure the relative concentration of proteins from plasma samples in relative fluorescent units (RFUs) and is described further in Supplementary Methods.

### Validation of SomaScan assay results with alternative protein analysis methods

We aimed to validate the aptamer analyses corresponding to 22 proteins and one protein complex that associated with the risk of hip fractures in the present study. We searched for studies in which the same patient blood samples were analyzed using both the SomaScan aptamer platform and an antibody-based proteomics platform. Only studies with more than 150 patient samples analyzed by both platforms were selected.

### Incident hip fractures

CHS participants self-reported hospitalizations every 6 mo, and Medicare claims data were used to identify hospitalizations not reported by participants. Following the 1992-1993 CHS study visit through 2015, incident hip fractures were identified from hospital discharge International Classification of Diseases, Ninth Revision (ICD9) codes 820.xx. Hospitalizations for pathologic fractures (ICD9 code 773.1x) and motor vehicle accidents (E810.xx–E825.xx) were excluded.

The hip fracture data for the HUNT participants were collected from the hospital-based registries in the region and cover the time interval from baseline (HUNT3 visit in 2006-2008) until March 2021. Hip fracture was defined as ICD10 codes S72.0, S72.1, S72.2, or ICD9 code 820.

### Statistical analyses

#### Cox regression

Associations between circulating protein levels (log transformed aptamer levels) and incident hip fractures were calculated for each cohort separately using Cox regression models, adjusting for age and sex. In CHS, adjustment for race (Black vs non-Black) was also included. As the HUNT subjects selected for proteomic analyses were a part of a HUNT cardiovascular project enriched in cardiovascular events, adjustment for incident cardiovascular event (Yes/No) was included in the HUNT analyses. In sensitivity analyses, sex-stratified Cox regressions were performed. Finally, for proteins associated with hip fracture risk in the meta-analysis, we performed sensitivity analyses restricting follow-up time to a maximum of 10 yr. The proportional hazards assumption was tested using scaled Schoenfeld residuals using the cox.zph function in R. Effect sizes are presented as hazards ratios (HR) per SD increase in log-transformed aptamer levels with 95% CIs.

#### Meta-analysis

The results from the CHS and the HUNT cohorts were combined using fixed effects IVW meta-analysis. Cochran’s test for heterogeneity was used to identify possible heterogeneity in the meta-analysis. Random effects meta-analysis (restricted maximum likelihood in the R package metafor) was used for aptamers with evidence of heterogeneity. To account for multiple testing, a conservative Bonferroni-adjusted *P*-value threshold of *P* < 1.0 × 10^−5^ (ie, *P* < 0.05/4979) was used to determine statistical significance. For assessing sex differences for proteins associated with hip fracture risk in the meta-analysis (*n* = 23), a *z*-test was used, adjusting the *P*-value threshold for the number of identified signals (*P* < .05/23 = 2.1 × 10^−3^). All statistical computations were performed using R.

#### Pathway analysis

To further understand biological mechanisms underlying associations between proteins of interest and hip fracture risk, we used ingenuity pathway analysis (IPA), a bioinformatics software that uses a manually curated database of protein associations, pathways, and known biological mechanisms to analyze and interpret omics data (QIAGEN; https://www.qiagenbioinformatics.com/products/ingenuity-pathway-analysis^30^^,^^31^). The input for the IPA analysis was a list of the 155 aptamers that passed a false discovery rate (FDR; threshold for a statistical significance of 0.10) and their direction of associations with incident hip fractures. The IPA software calculates one-sided *P*-values using a right-tailed Fisher’s exact test to estimate the probability that the top hits identified from association analyses overlap due to chance with proteins the software classified within a particular canonical pathway. *P*-values were adjusted using a Benjamini–Hochberg multiple testing correction, which is the default within the IPA software. Additionally, to estimate the direction of association between top hits and canonical pathways, the software calculates a *z*-score based on the known directional effect of each molecule on the pathway.

#### Mendelian randomization and colocalization

We used Mendelian randomization (MR) to assess possible causal effects of identified hip fracture-associated circulating proteins on fracture risk (using previously published summary statistics from Trajanoska et al.; 37 857 cases with fracture at any bone site and 227 116 controls^32^) and estimated bone mineral density (eBMD) measured by quantitative heel ultrasound (using previously published summary statistics from Morris et al.; 426 824 individuals^33^) along with summary statistics of protein quantitative trait loci (pQTLs) from the deCODE (*n* = 35 559; Ferkingstad et al.^34^) and Fenland (*n* = 10 708; Pietzner et al.^35^) cohorts as source for genetic instruments of the exposure. The primary analysis was done using the genetic instruments (*cis*-pQTLs) from the deCODE cohort^34^ for protein levels. Replication analyses, using an alternative set of pQTLs, were performed using genetic instruments derived from the Fenland study.^35^ To ensure that the instruments for protein levels were independent, we performed linkage disequilibrium (LD)-clumping (pair-wise LD *r*^2^ < 0.001), using a random 5000-individual reference panel from the UK biobank (European ancestry). The primary MR results were based on the Wald-ratio (1 genetic instrument), or IVW (IVW ≥ 2 instruments) methods. For proteins that had ≥3 instruments, we also performed sensitivity analyses using weighted-median, weighted-mode, and MR-Egger. For circulating proteins with evidence of potential causal effects on eBMD or fracture, we performed colocalization analyses using the coloc R package with default prior settings (*P*1 = *P*2 = 1 × 10^−4^, *P*12 = 1 × 10^−5^). A posterior probability (PP) of the shared causal variant hypothesis *H4* > 0.8 (*H4*: both traits are associated and share the same single causal variant) was considered strong colocalization evidence, ie, the genetic signals are shared by the circulating protein level and eBMD. To exclude reverse causality, wherein eBMD influences circulating levels of GHR or CHRDL1, we performed MR using a genetic risk score for eBMD^33^^,^^36^ as the instrument and circulating levels of GHR or CHRDL1 (evaluated in the HUNT cohort, *n* = 3188) as the outcomes.

## Results

Participant characteristics in CHS and HUNT are summarized in Table 1. CHS had older participants (mean age of 74.4 yr compared to 64.5 yr in HUNT) and fewer men (39% compared with 61%) than HUNT.

### Protein levels in the populations

**Figure 1:**
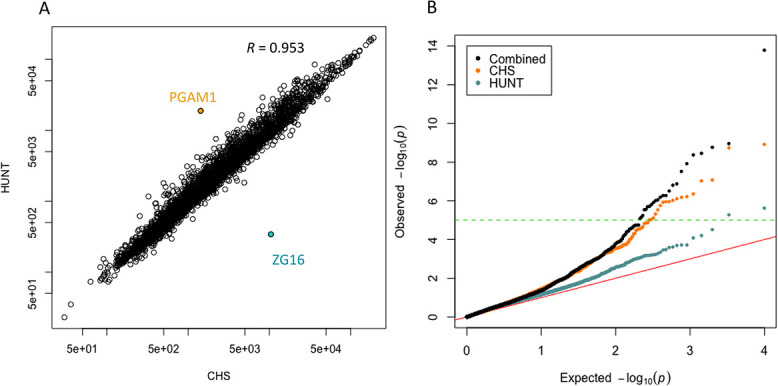
Correlation between protein levels and QQ plot for CPAS analyses. (A) Correlation between protein levels in CHS (*x*-axis) and HUNT (*y*-axis), with log scaled axes. Each point corresponds to the mean relative fluorescent unit (RFU) of one of the 4979 aptamers in all individuals. *R* is the Pearson correlation coefficient. (B) QQ plot of observed Cox regression *P*-values (*y*-axis) vs expected null model *P*-values (*x*-axis) for each cohort, and for the meta-analysis. Negative log *P*-values are shown. The red line shows expected result if no associations exist. The green dotted line indicates the Bonferroni threshold (*P* = 1 × 10^−5^). CHS, Cardiovascular Health Study; CPAS, Circulating Proteome Association Study; HUNT, Trøndelag Health Study; QQ, quantile–quantile.

Summary statistics for the plasma levels (expressed as RFU) of all 4979 evaluated aptamers were computed for CHS and HUNT (Supplementary Table S1, Figure 1A, Supplementary Figure S1). Mean aptamer values in the two cohorts were well correlated except for two aptamers, corresponding to the ZG16 and PGAM1 proteins (Figure 1A). Expression levels of ZG16 differed strongly in Black and non-Black participants (Supplementary Figure S1B). The reason for the higher levels of PGAM1 in HUNT compared with CHS is unclear. However, pre-analytic variability due to differences in storage and temperature can result in differences in enzymatic activity in blood/plasma samples, and PGAM1 has previously been observed to be sensitive to sample handling conditions.^37^ Five well-known sex-specific proteins were differentially expressed between men and women in both cohorts (higher expression in women in both cohorts of LHB, FSH, HCG, and PZP; higher expression of PSA in men in both cohorts; Supplementary Figure S2).

### Association with incident hip fracture

The 3171 CHS participants experienced 456 incident hip fractures during a mean follow-up of 12.6 yr, and the 3259 HUNT participants experienced 187 incident hip fractures during a mean follow-up of 11.5 yr (Table 1). The average (SD) follow-up times until incident hip fracture were 12.6 (6.3) yr and 7.5 (3.7) yr in CHS and HUNT, respectively. The association results between aptamers and incident hip fractures from the CHS cohort and the HUNT cohort were meta-analyzed (Figure 1B). A quantile–quantile (QQ) plot of the results from the CPAS meta-analysis and the CPAS performed in each individual cohort illustrates that the meta-analysis yielded more significant associations than either individual study (Figure 1B).

**Figure 2:**
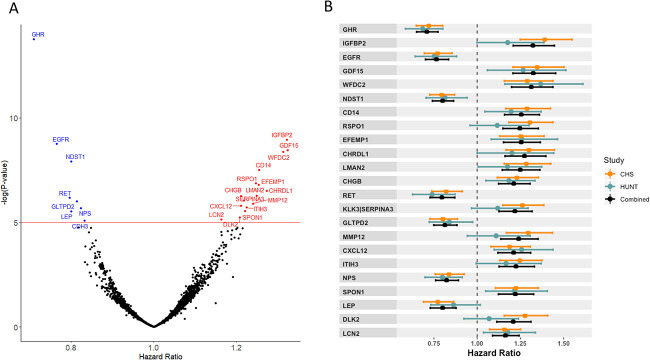
Proteins associated with incident hip fracture risk. (A) Volcano plot of the results from the Circulating Proteome Association Study (CPAS) meta-analysis, with hazard ratios on the *x*-axis and negative log *P*-values on the *y*-axis. The red line marks the Bonferroni significance level *P* = 1 × 10^−5^. Aptamers above the significance line in blue were associated with a reduced hip fracture risk, and those above the significance line in red were associated with an increased hip fracture risk. (B) The proteins corresponding to the 23 aptamers most significantly associated with incident hip fracture. HR, hazard ratio, error bars show 95% CIs. Results are shown for the CHS cohort (orange), the HUNT cohort (blue), and combined cohorts (black). CHS, Cardiovascular Health Study; HUNT, Trøndelag Health Study.

The associations between the 4979 evaluated aptamers and hip fracture risk are shown in a volcano plot in Figure 2A. We identified 23 aptamers that were significantly associated with incident hip fractures (Bonferroni correction 0.05/4979, *P* < 1.0 × 10^−5^), corresponding to 22 proteins (*increased risk*: IGFBP2, GDF15, WFDC2, CD14, RSPO1, EFEMP1, CHRDL1, LMAN2, CHGB, MMP12, CXCL12, ITIH3, SPON1, DLK2, and LCN2; *decreased risk*: GHR, EGFR, NDST1, RET, GLTPD2, NPS, and LEP) and one protein complex (*increased risk:* KLK3/SERPINA3 complex). For all 23 aptamers, the observed effect sizes were generally similar in CHS and HUNT (Figure 2B, Supplementary Table S2). When evaluated using the scaled Schoenfeld residual test in the separate cohorts, there was no evidence of violation of the proportional hazard assumption for any of the 23 identified hip fracture-associated aptamers. Moreover, the assay precision data for each of the 23 individual proteins were excellent for all SomaScan assays, except for LCN2 (Supplementary Table S3).

The strongest associations with decreased hip fracture risk were observed for the aptamer targeting the extracellular part of the growth hormone receptor (GHR; HR per 1 SD higher log transformed protein levels 0.71; 95% CI, 0.65–0.77, *P* = 1.6 × 10^−14^), reflecting the levels of the proteolytically cleaved extracellular part of the GHR called the GH-binding protein, and the aptamer for the soluble epidermal growth factor receptor (EGFR, HR 0.77; 95% CI, 0.70–0.83, *P* = 1.7 × 10^−9^; Figure 2A and B, Table 2, Supplementary Table S2). The strongest associations with increased hip fracture risk were observed for aptamers for insulin-like growth factor binding protein 2 (IGFBP2, HR 1.32; 95% CI, 1.21–1.45, *P* = 1.1 × 10^−9^) and growth differentiation factor 15 (GDF15 or MIC-1*,* HR 1.32; 95% CI, 1.21–1.45, *P* = 3.5 × 10^−9^).

**Table 2:** Proteins statistically significantly associated with hip fracture risk in the meta-analysis.

		**Meta-analysis**			
**Entrez gene name**	**Full name**	**HR**	**95% CI low**	**95% CI high**	***P*-value**
*GHR*	Growth hormone receptor	0.71	0.65	0.77	1.6E-14
*IGFBP2*	Insulin-like growth factor-binding protein 2	1.32	1.21	1.45	1.1E-09
*EGFR*	Epidermal growth factor receptor	0.77	0.70	0.83	1.7E-09
*GDF15*	Growth/differentiation factor 15	1.32	1.21	1.45	3.5E-09
*WFDC2*	WAP four-disulfide core domain protein 2	1.31	1.20	1.44	4.2E-09
*NDST1*	Bifunctional heparan sulfate N-deacetylase/N-sulfotransferase 1	0.80	0.74	0.86	1.2E-08
*CD14*	Monocyte differentiation antigen CD14	1.26	1.16	1.36	3.0E-08
*RSPO1*	R-spondin-1	1.25	1.15	1.35	1.3E-07
*EFEMP1*	EGF-containing fibulin-like extracellular matrix protein 1	1.25	1.15	1.37	1.5E-07
*CHRDL1*	Chordin-like protein 1	1.27	1.16	1.40	3.1E-07
*LMAN2*	Vesicular integral-membrane protein VIP36	1.25	1.15	1.36	5.3E-07
*CHGB*	Secretogranin-1	1.21	1.12	1.31	5.3E-07
*RET*	Proto-oncogene tyrosine-protein kinase receptor Ret	0.80	0.73	0.87	6.5E-07
*KLK3|SERPINA3*	Alpha-1-antichymotrypsin complex	1.22	1.13	1.32	8.8E-07
*GLTPD2*	Glycolipid transfer protein domain-containing protein 2	0.81	0.75	0.88	9.8E-07
*MMP12*	Macrophage metalloelastase	1.24	1.14	1.35	1.2E-06
*CXCL12*	Stromal cell-derived factor 1	1.21	1.12	1.31	1.6E-06
*ITIH3*	Inter-alpha-trypsin inhibitor heavy chain H3	1.22	1.13	1.33	1.9E-06
*NPS*	Neuropeptide S	0.82	0.76	0.89	2.0E-06
*SPON1*	Spondin-1	1.22	1.12	1.33	2.8E-06
*LEP*	Leptin	0.80	0.73	0.88	2.9E-06
*DLK2*	Protein delta homolog 2	1.21	1.11	1.31	5.7E-06
*LCN2*	Neutrophil gelatinase-associated lipocalin	1.16	1.09	1.24	7.0E-06

The association between aptamers and fracture risk was determined by Cox regression in the two cohorts separately, and the results were then combined using fixed effect inverse-variant meta-analysis. A Bonferroni *P*-value threshold of 0.05/4979 = 1 × 10^−5^ is used. CI, confidence interval; HR, hazard ratio

When stratified by sex, associations between the 23 identified aptamers and hip fracture risk were not meaningfully different between men and women (Supplementary Figure S3, Supplementary Table S4). The mean follow-up time among all participants was 12.1 yr (22 yr maximum follow-up), giving a total follow-up time of 77 513 person-years. Limiting participant hip fracture follow-up time to 10 yr (corresponding to the time used for fracture prediction in the clinically frequently used Fracture Risk Assessment Tool: FRAX^38^) did not materially change the strength of the association for any of the 23 identified aptamers with hip fracture risk (Supplementary Figure S4, Supplementary Table S5). For each study, correlations among the circulating levels of the 23 identified hip fracture-associated proteins are shown as heat maps in Supplementary Figure S5. As expected, some of the proteins were strongly correlated, suggesting that their associations with hip fracture risk may reflect the same biological phenomenon. In both cohorts, the strongest correlation was observed between GHR and IGFBP2, which both are part of the GH/IGF system. High GHR levels were associated with low IGFBP2 levels (Supplementary Figure S5).

### Replication of previously reported associations for proteins with hip fracture risk

When Nielson et al. evaluated 379 circulating proteins in a subsample of the Osteoporotic Fractures in Men (MrOS) cohort, including 129 hip fracture cases, five proteins were associated with an increased risk of hip fractures.^39^ In the present study, two of these five proteins (CD14 and CHL1) were replicated with statistically significant associations in the same direction as observed in the previous MrOS cohort (Supplementary Table S6). High CD14 levels were associated with increased hip fracture risk in both MrOS and the present study. As the statistical significance of the association (*P* = 3.0 × 10^−8^) passed the conservative Bonferroni threshold in the present study, CD14 was among our 23 identified proteins (Figure 2, Table 2, Supplementary Table S2). High CHL1 levels were also associated with increased risk of incident hip fractures in the present study, but the statistical significance of evidence for this association was moderate (*P* = 1.7 × 10^−3^, Supplementary Table S6). Two other proteins, C7 and A2M, were not replicated in the present study and the fifth protein, FCGBP, was not included in the present CPAS meta-analysis (Supplementary Table S6).

### Validation of the SomaScan assays with alternative protein analysis methods for the 23 identified proteins

We identified four studies^21^^,^^34^^,^^40^^,^^41^ validating SomaScan assays with the antibody-based proteomics platform Olink. Out of the top 23 SOMAmers identified in the present study, 12 were evaluated in at least one of these studies.^34^^,^^40^^,^^41^ Nine out of these 12 proteins had a mean correlation between platforms of r > 0.70 (IGFBP2, GDF15, WFDC2, RSPO1, RET, MMP12, SPON1, LEP, and LCN2), while the remaining three had a correlation (r) between 0.55 and 0.70 (EGFR, EFEMP1, and CHRDL1; Supplementary Table S7).

In addition, we found that the specificity of nine of the SOMAmers for our top 23 hits was previously validated by a qualitative mass spectrometry (MS) technique (IGFBP2, EGFR, WFDC2, CD14, CHRDL1, CHGB, CXCL12, ITIH3, and LEP; Supplementary Table S7).^23^ The MS validation was done by enriching proteins with SOMAmers, and then the SOMAmer-protein complexes were purified and sequenced by MS. We did not find validation for seven SOMAmers by either Olink or qualitative MS, but five had an inferred validation by *cis*-pSNP (protein single-nucleotide polymorphisms: *GHR*, *NDST1*, *LMAN2*, *GLTPD2*, and *DLK2*; Supplementary Table S7),^23^ supporting that the used SOMAmer targets the correct protein. We did not identify any studies evaluating the validity of the NPS or KLK3/SERPINA3 SOMAmers (Supplementary Table S7).

### Pathway analysis

**Figure 3:**
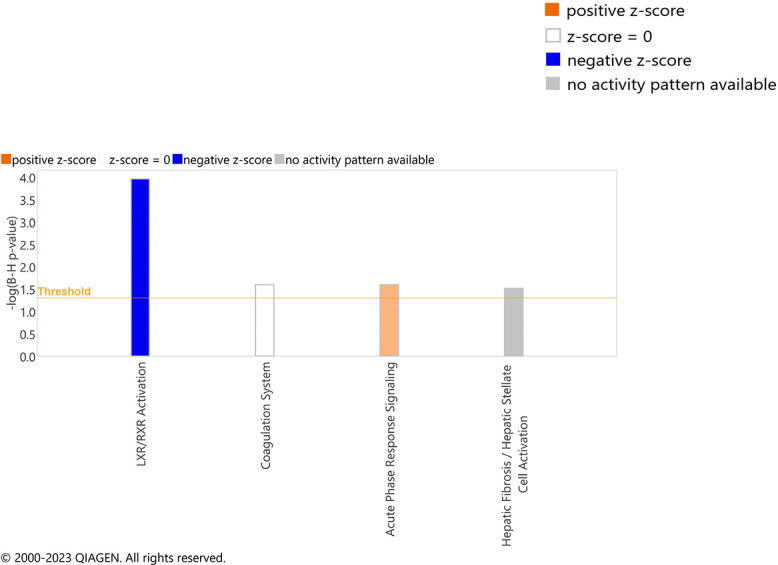
Ingenuity pathway analysis (IPA). The input for the IPA was a list of 155 top hit aptamers, passing a false discovery rate threshold for significance of 0.10, and their direction of associations with incident hip fractures (Supplementary Table S8). The IPA software calculates one-sided *P*-values using a right-tailed Fisher’s exact test to estimate the probability that the overlap between the top hits identified from association analyses and proteins that the software has classified within a particular canonical pathway are due to random chance. *P*-values given in the figure are adjusted using a Benjamini–Hochberg multiple testing correction. A *z*-score is additionally calculated to estimate the direction of association between top hits and canonical pathways based on the known directional effect of each molecule on the pathway.

To identify pathways associated with hip fracture risk, we included the top 155 aptamers with an FDR < 0.10 from the CPAS meta-analysis in a pathway analysis using IPA. This analysis identified the canonical LXR/RXR activation pathway to be most strongly associated with hip fracture risk, with a negative *z*-score suggesting downregulation of the pathway among those with increased hip fracture risk (Figure 3, Supplementary Table S8). Additionally, pathway analyses identified the coagulation system and acute phase response signaling. For the acute phase response signaling pathway, a positive *z*-score was observed, suggesting upregulation of the pathway among those with increased hip fracture risk (Figure 3).

### Mendelian randomization

We next aimed to determine whether the 23 circulating hip fracture-associated proteins had causal effects on fracture risk and/or BMD using MR. We identified valid genetic instruments for 20 of these circulating proteins to be used in *cis*-pQTL-based MR that evaluated the associations with fracture^32^ and eBMD^33^ outcomes. We used such *cis*-pQTL signals, because they are less likely to be influenced by horizontal pleiotropy. The main results are reported for analyses using genetic instruments derived from the large deCODE pQTL SomaScan dataset.^34^ For statistically significant findings, we also successfully replicated the results using genetic instruments from the Fenland pQTL dataset.^35^ After adjustment for 39 multiple comparisons (*P* < 0.05/39 = 1.3 × 10^−3^; 20 comparisons for eBMD and 19 comparisons for fracture), we observed that increased genetically determined CHRDL1 levels were associated with increased eBMD (0.062 SD, SE 0.011, *P* = 7.1 × 10^−9^ per SD increase in CHRDL1; Supplementary Figure S6A; Supplementary Table S9) in MR findings. We also observed that increased genetically determined GHR levels were associated with reduced eBMD (−0.067 SD, SE 0.010, *P* = 1.2 × 10^−11^ per SD increase in GHR; Supplementary Figure S6A; Supplementary Table S9) in MR findings. We did not find evidence for causal associations with fractures for any of the evaluated proteins with valid genetic instruments (Supplementary Table S9). For the CHRDL1 signal, there was strong evidence of colocalization between pQTL and eBMD (Supplementary Figure S6B, PP.H4 = 99.9%). For the top *GHR* signal, rs4610468, there was no evidence of colocalization between pQTL and eBMD (Supplementary Figure S6C; PP.H4 < 0.1%), which suggested that the MR-identified association may be confounded by LD or that multiple independent colocalized signals exist in this region. Interestingly for both these proteins, the estimated causal association with eBMD was in the opposite direction to what was expected based on the observational association with hip fractures.

To assess the influence of reverse causality, wherein eBMD influences circulating levels of GHR or CHRDL1, we performed MR using a genetic risk score for eBMD^33^^,^^36^ as the instrument and circulating levels of GHR or CHRDL1 (evaluated in the HUNT cohort, *n* = 3188) as the outcomes. This provided no evidence that genetically determined eBMD was causally related to circulating GHR or CHRDL1 levels (*P* > 0.05 for both *GHR* and *CHRDL1*).

## Discussion

Although hip fractures are common and associated with major disability and mortality, the exact biological mechanisms that underlie susceptibility to hip fractures remain incompletely understood. To identify novel protein biomarkers for hip fractures, we performed a CPAS meta-analysis, which identified 23 proteins that were statistically significantly associated with incident hip fractures, including several inflammation-related proteins. In addition, pathway analysis identified reduced LXR/RXR activation and increased acute phase response signaling to be overrepresented among those with increased hip fracture risk.

There was a high correlation of the average expression of the different aptamers between CHS and HUNT, and the strengths of the associations for the 23 identified proteins with hip fracture risk were similar. To our knowledge, this is the first comprehensive and robust evaluation of the proteome in hip fracture. Although a previous hip fracture GWAS meta-analysis including 11 516 hip fracture cases only identified five genetic signals for hip fractures,^9^ the present study, using CPAS meta-analyses of a data set including 643 incident hip fracture cases, identified 23 proteins significantly associated with hip fractures. These findings suggest that the CPAS meta-analytic approach is efficient to identify hip fracture signals that may be used to enhance the understanding of the biological mechanisms underlying hip fractures or for future studies evaluating hip fracture prediction. The strength of CPAS meta-analyses might be due to the fact that proteins regulate biological processes and can integrate the effects of genes with those of the environment, age, comorbidities, behaviors, and drugs.^24^ Future large-scale meta-analyses of proteomics data including a higher number of incident hip fractures might identify additional proteins associated with hip fractures.

This is an observational study, and we are not able to distinguish whether the proteins that are associated with incident hip fractures are causal, if they are correlated with another unobserved factor that are causal of hip fractures or if they are just an indicator of current health status. In the Supplementary Discussion, we discuss what is currently known about the 23 proteins associated with hip fractures and how they could affect hip fractures.

In pathway analyses, downregulation of the LXR/RXR activation pathway was the most strongly associated with increased hip fracture risk. LXR and RXR are nuclear receptors that, upon ligand activation of either receptor, can form LXR/RXR heterodimers, which can activate transcription of target genes.^42^ A role of this pathway for bone mass regulation is supported by the fact that LXR/RXR heterodimers can block osteoclast differentiation.^43^ In addition, both the synthetic LXR agonist T0901317 and the synthetic RXR agonist bexarotene protect mice from ovariectomy (ovx)-induced bone loss.^43^^,^^44^ Ovx mice treated with the LXR agonist had fewer osteoclasts due to a reduced RANKL/OPG (receptor activator of NF-κB ligand/osteoprotegerin) ratio.^44^ Both LXR and RXR exert anti-inflammatory effects,^45^^,^^46^ and treating mice with an LXR agonist protected mice from inflammatory-induced bone loss.^45^ Collectively, these findings indicate that LXR/RXR activation is an interesting target to improve bone health.

Besides the LXR/RXR activation pathway, IPA revealed that both the acute phase response pathway and the coagulation pathway were associated with hip fracture risk in the present study. These two pathways are activated by inflammation and tissue injury. Daily movements cause microinjuries in the musculoskeletal system, which triggers the acute phase response and coagulation systems to initiate normal tissue repair. During aging, these systems are dysregulated, potentially leading to a persistent cycle of acute phase response that cause degeneration of the musculoskeletal tissues.^47^

The SOMAmers are complementary to the shape of the target protein and have been shown to be very specific as they can distinguish between closely related proteins and protein isoforms.^48^ However, this could also be a disadvantage, as only one SNP could lead to an amino acid substitution, which could alter the electric charge and conformation (ie, shape) of the protein. Consequently, the aptamer may not have the same binding affinity or may not recognize its target protein’s tertiary structure.^49^ To validate binding between the SOMAmers identifying our 23 top hits and the intended protein, we searched for studies using orthogonal methods. Out of the 23 aptamers associated with the risk of hip fractures in the present study, we found evidence from the immune-based Olink assay, mass spectrometry, and/or *cis*-pSNPs that 21 of them bind to the correct target protein.^21^^,^^23^^,^^34^^,^^40^^,^^41^ Collectively, we believe that there is strong evidence that most of the circulating proteins identified in the present study were associated with hip fracture risk and that they were accurately quantified using the aptamer-based assays.

The study has limitations. Although the SomaScan platform used in our study analyzed almost 5000 proteins, this is only about 25% of the proteins that have been identified by the Human Proteome Project.^50^ SomaScan measures the relative but not absolute concentration, which prevents direct comparisons with results obtained by other techniques. Not all aptamers used to find proteins associated with incident hip fractures have been validated by other techniques, and we cannot be certain that all proteins in our study have been correctly bound by their specific SOMAmer. Although all hip fractures included in the present meta-analyses were incident hip fractures occurring after the collection of the baseline samples for proteomics, it is a limitation that information on non-hip fractures was not available. Confounding, measurement error, and selection bias cannot be excluded in observational studies. However, this study has some important strengths. A major strength is the high number of incident hip fracture cases included. Also, both cohorts were analyzed using the same SomaScan version 4.0, one of the most comprehensive high throughput proteomic assays available, likely contributing to the high correlation of the average expression of the different aptamers between the two cohorts.

In conclusion, we identified several circulating proteins and pathways consistently associated with incident hip fractures. These findings support the usefulness of the CPAS meta-analytic approach for comprehensive proteomic studies in a similar manner as has previously been observed for the meta-analytic approach in human genetic studies. Additionally, the CPAS meta-analytic approach provides a more detailed picture of biological processes underlying susceptibility to hip fracture risk. Future studies should investigate the underlying biology of these potential novel drug targets. In addition, future studies should determine the clinical utility of protein-based risk scores for hip fracture prediction.

## Supplementary Material

Austin_et_al_SupplFigures_zjad011

Suppl_Tables_zjad011

Suppl_Material-Methods_and_Discussion_zjad011

## Data Availability

Individual level data from HUNT can be accessed by, or in collaboration with, a Norwegian principal investigator. Researchers can apply for HUNT data access from HUNT Research Centre (https://www.ntnu.edu/hunt) if they have obtained project approval from the Regional Committee for Medical and Health Research Ethics (REC). Information on the application and conditions for data access is available at https://www.ntnu.edu/hunt/data. For cohort-specific data requests of CHS, contact Tom Austin (austintr@uw.edu).
