# Supplementary figures and images for: Large-scale circulating proteome association study (CPAS) meta-analysis identifies circulating proteins and pathways predicting incident hip fractures
Source: J Bone Miner Res. Author manuscript; Available in PMC 2025 Mar 22. (PMC11070286; doi:10.1093/jbmr/zjad011)

Fig. S1

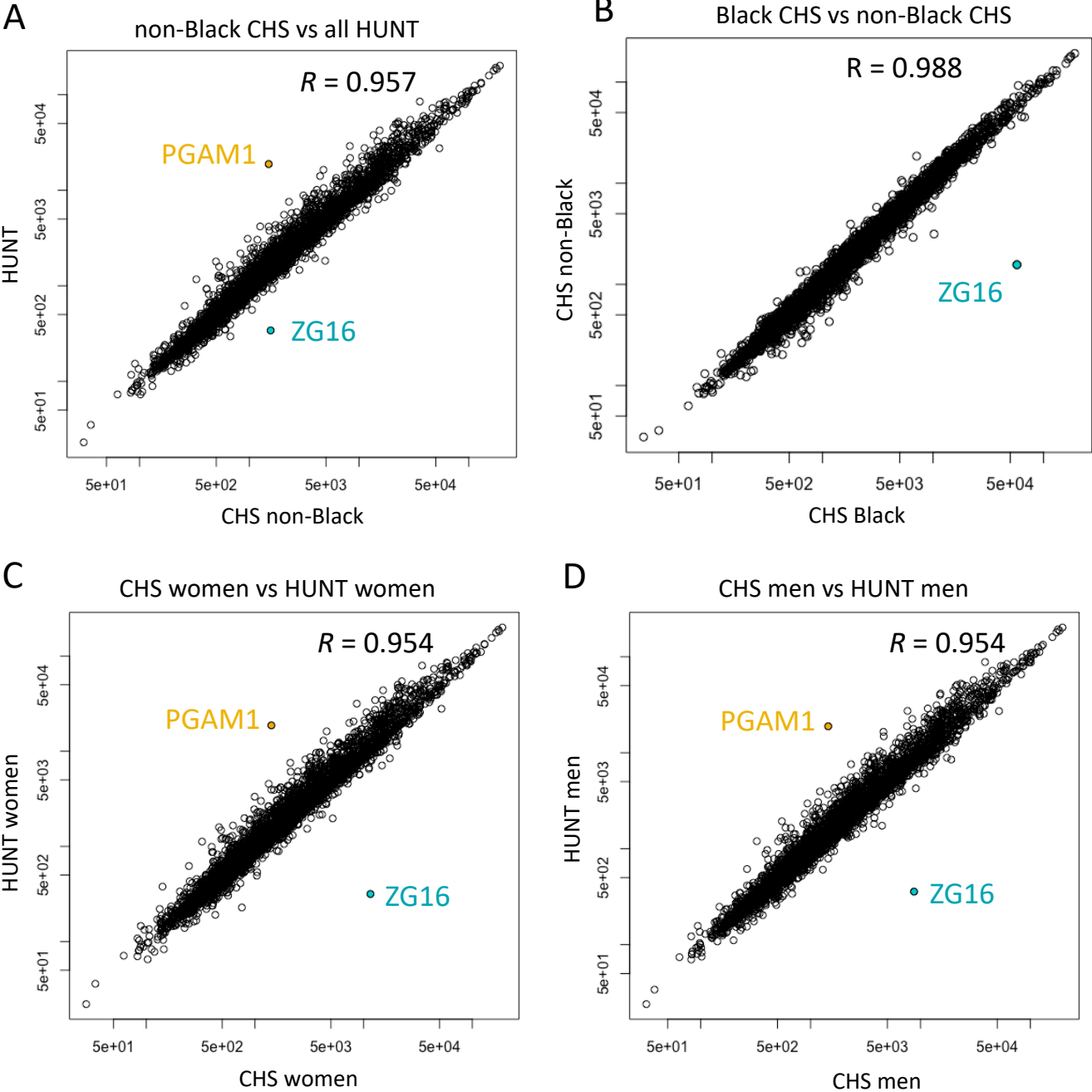

Fig. S2

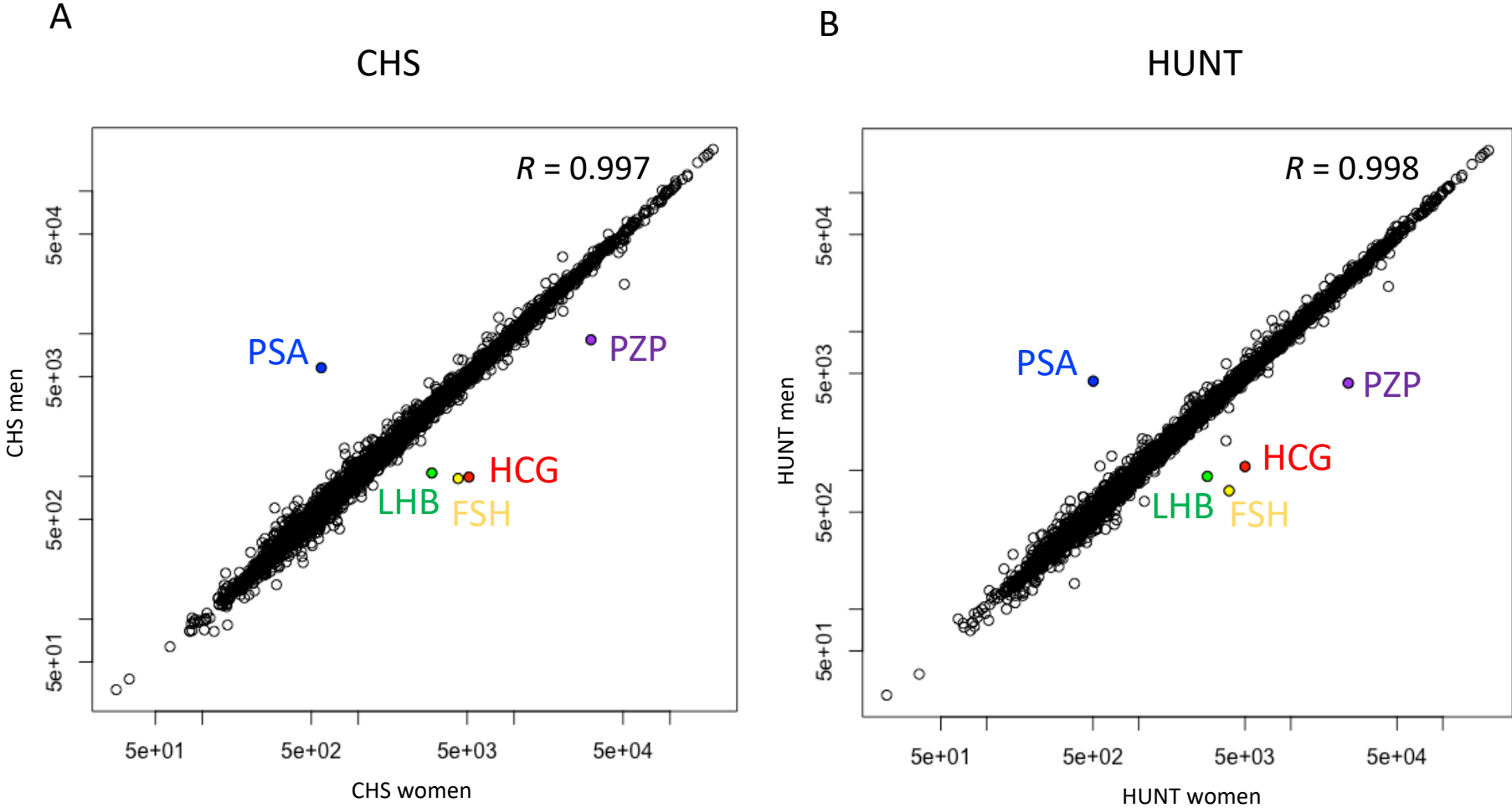

Fig. S3

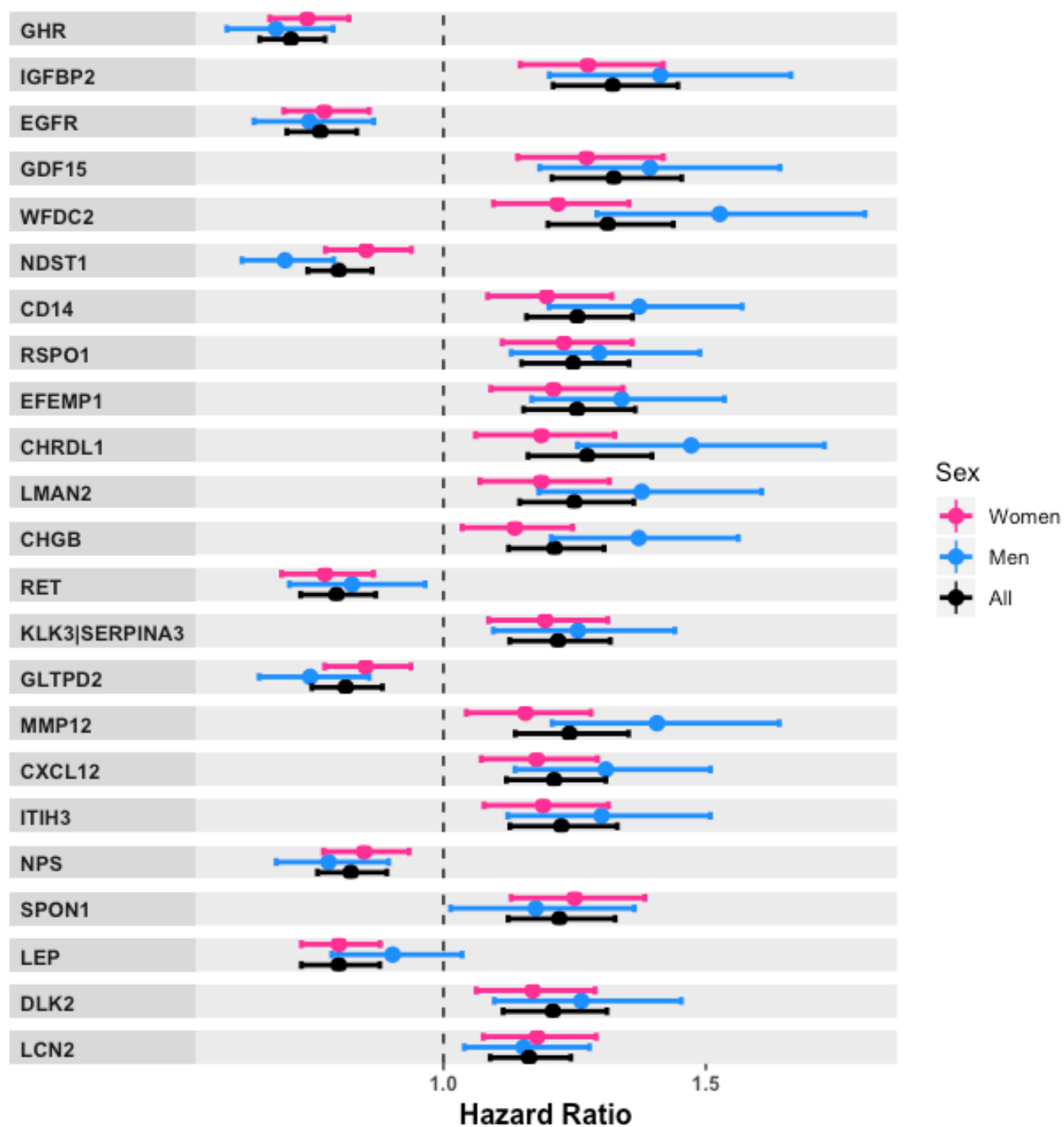

Fig. S4

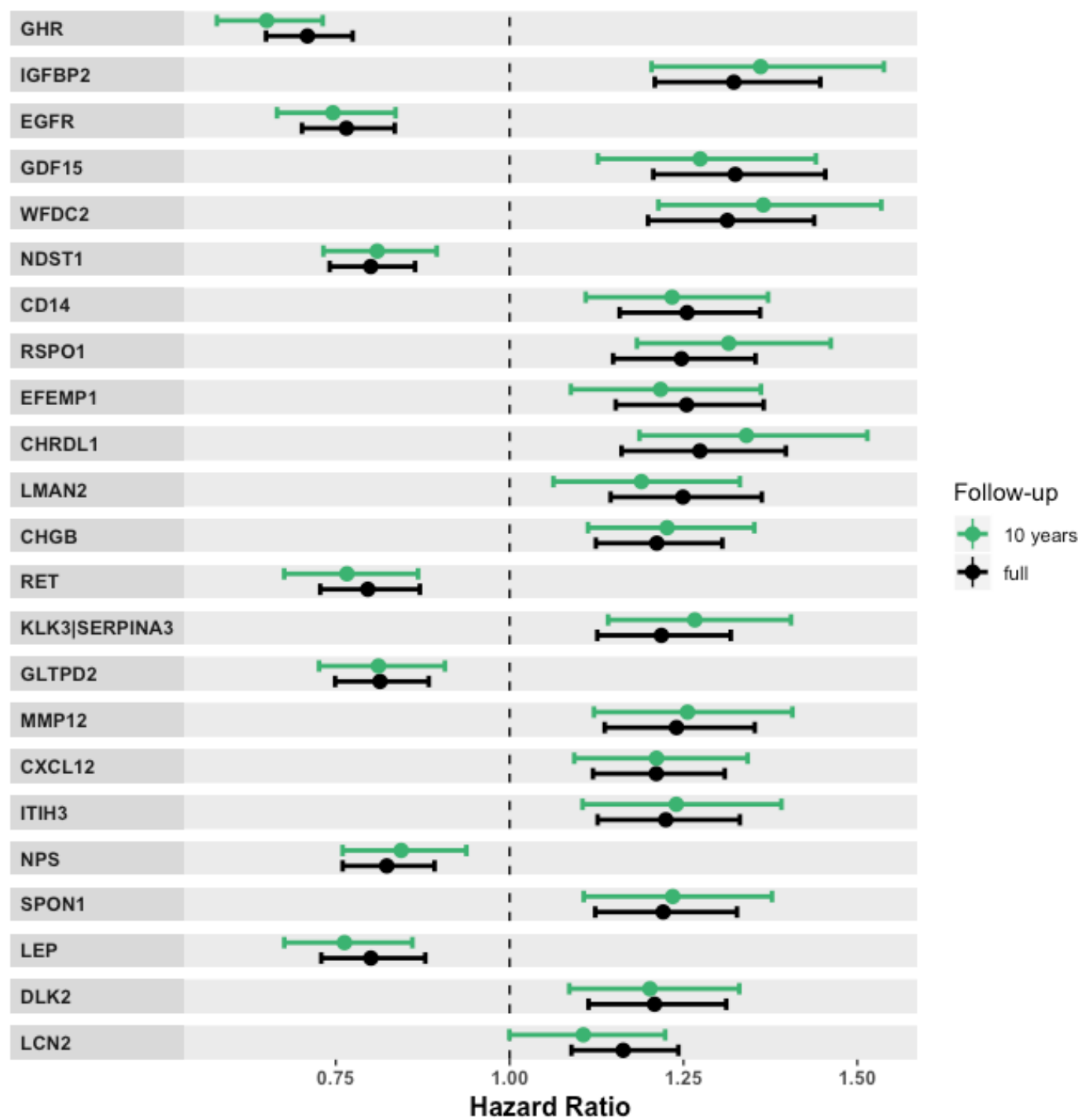

Fig. S5

HUNT

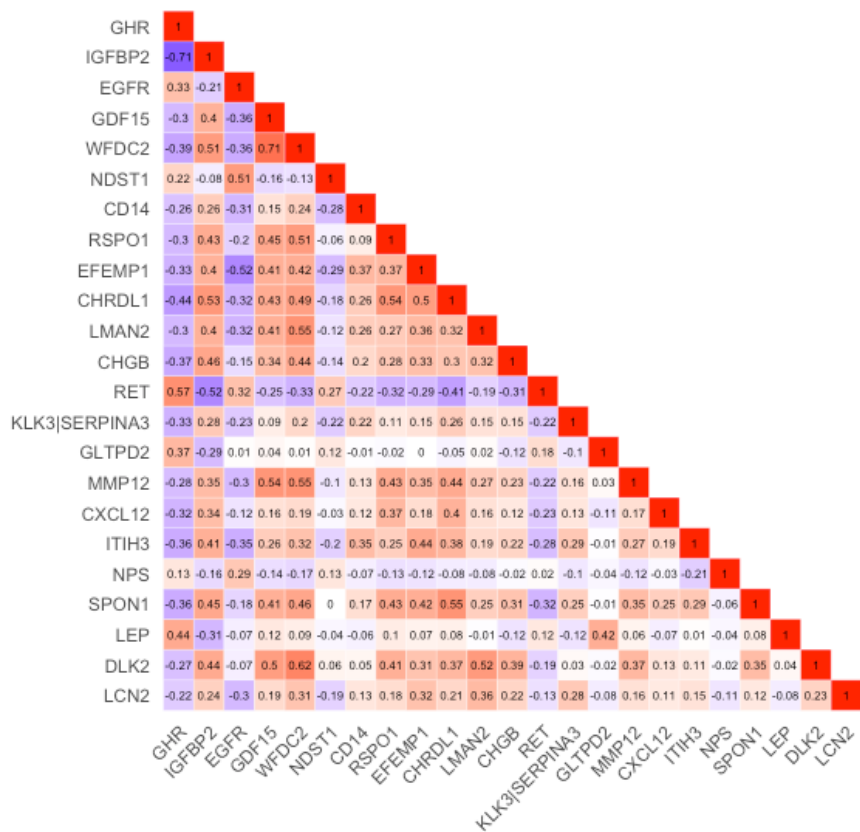

CHS

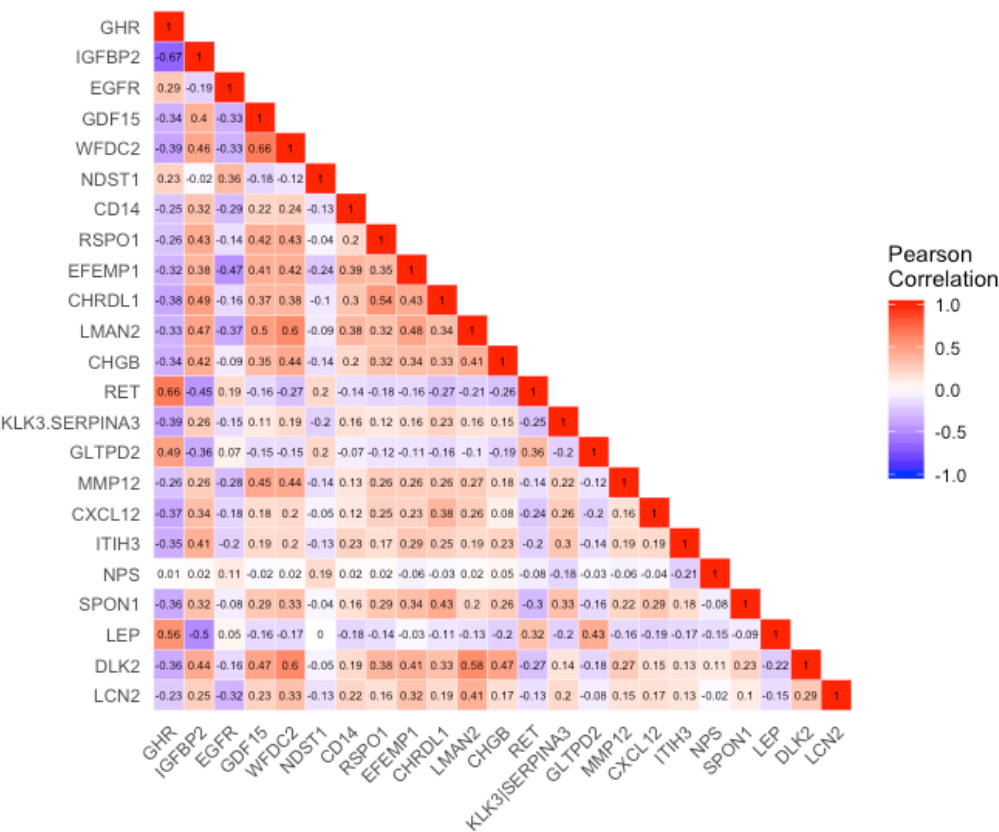

Fig. S6

A

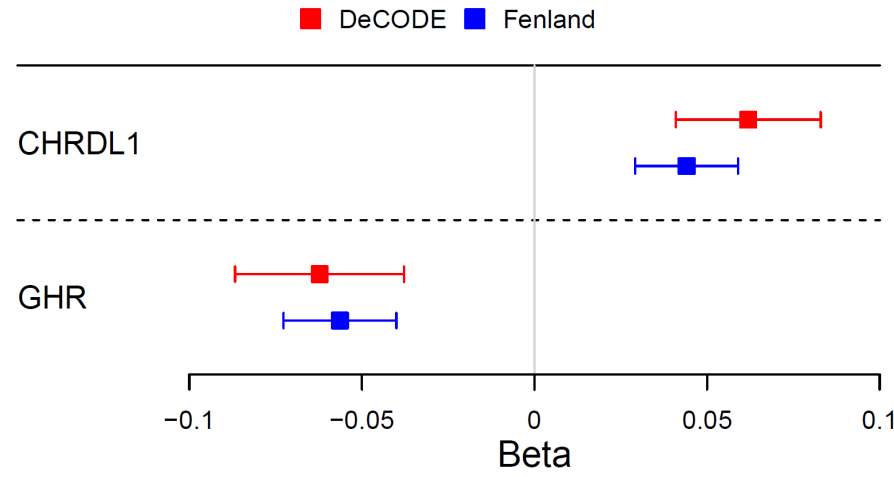

B

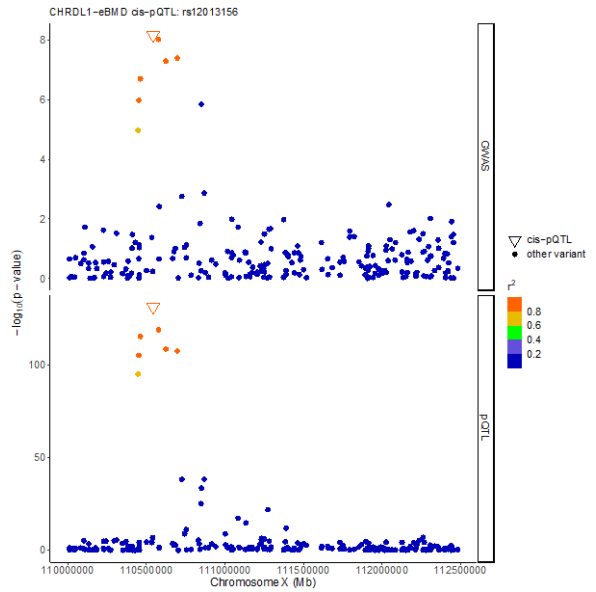

C

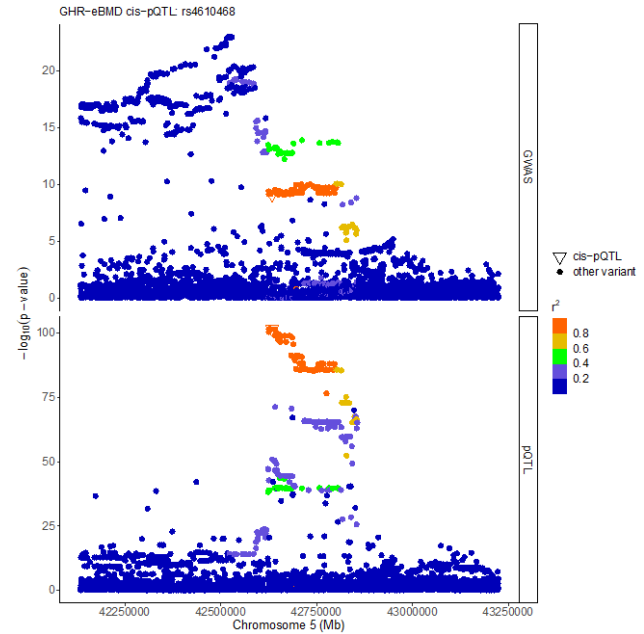

Supplement: Suppl. figures S1-S6 [file NIHMS1983409-supplement-Suppl__figures_S1-S6.pdf]
