# Supplementary material for: Large-scale circulating proteome association study (CPAS) meta-analysis identifies circulating proteins and pathways predicting incident hip fractures
Source: J Bone Miner Res. 2024 Jan 4;39(2):139–49. doi: 10.1093/jbmr/zjad011 (PMC11070286; doi:10.1093/jbmr/zjad011)
Supplement: Supplementary file 1 [file Suppl_Material-Methods_and_Discussion_zjad011.docx]

# **Large-scale Circulating Proteome Association Study (CPAS) meta-analysis identifies circulating proteins and pathways predicting incident hip fractures**

# **Supplementary Material**

# **Methods**

## Proteomics

Briefly, the aptamers from SomaScan are single-stranded DNA-based reagents called SOMAmers (Slow Off-rate Modified Aptamers). The negatively charged SOMAmers are designed to be complementary to the shape of the natively folded target proteins and bind the target protein tightly and specifically at a ratio of 1:1 ^1,2^. The method, which takes advantage of novel chemically modified nucleotides, converts the measurement of protein levels into the measurement of nucleic acid levels assessed by a DNA oligo-array plate reader ^1,3^. The assay sensitivity has a median lower limit of detection in the femtomolar range, which is comparable to that of typical immunoassays ^4,5^. Results of these assays, reported in RFU, are approximately proportional to plasma protein concentrations. Median intra- and inter-assay coefficients of variation for SomaScan version 4.0 are low, ~5% ^4,5^. The assay used for these analyses includes 5,284 aptamers. We excluded aptamers marked as “deprecated” (indicating a retired aptamer) and those marked as “non-human” from the present analyses. Samples flagged by SomaLogic for poor quality assay were also removed. The remaining 4,979 aptamers, corresponding to 4,860 unique proteins were included in our analyses (Table S1). 

# **Discussion**

## **Current knowledge about the 23 proteins associated with hip fractures**

The strongest association with hip fracture risk was observed for circulating GHR. A large proportion of circulating GH is bound to the GHBP, which in humans is the proteolytically cleaved extracellular, ligand binding part of the transmembrane GHR^6^. The SOMAmer reagent for GHR targets amino acids 27–264 of the GHR, and the extracellular domain of GHR includes amino acids 1-246^7^. Thus, it is highly likely that the abundant circulating GHBP is the main source of the identified circulating GHR signal. We observed that high GHR/GHBP levels are associated with substantially reduced hip fracture risk. This finding is in line with previous studies revealing increased fracture risk in hypopituitary patients with GH-deficiency and that GH treatment increases BMD in these patients^8,9^. Patients with GH-deficiency receiving GH treatment also had a lower fracture risk than patients left untreated^10^. In addition, it has been reported that GH treatment increases bone mineral content in postmenopausal women^11^. High circulating IGF binding protein 2 (IGFBP2), a known inhibitor of IGF action, was in the present study associated with an increased risk of hip fractures. In line with this, GH treatment decreases the serum levels of IGFBP2^12^, and IGFBP2 levels were increased in osteoporotic patients with fractures compared to non-osteoporotic controls^13^. 

Similar to GHR, the extracellular domain of the transmembrane EGFR can be proteolytically cleaved and released to the circulation^14^. In the present study, high circulating EGFR associated with a decreased risk of hip fractures. This is consistent with the previous findings that suppressing EGFR signaling in old mice accelerates cortical bone loss^15^ and that mice lacking the EGFR display osteopenia^16^. EGFR is bound by the EGF-containing fibulin-like extracellular matrix protein 1 (EFEMP1), thereby inhibiting the interaction between EGF and the EGFR^17-21^. This inhibition may be the reason why high circulating EFEMP1 levels associated with an increased risk of hip fractures in the present study. A possible role of EFEMP1 for bone health is supported by the fact that a genetic variant in the *EFEMP1* locus associates with cortical bone area in humans and that EFEMP1 deficient mice have increased cortical bone thickness^22^.

GDF15 is a stress-inducible cytokine, which is known to reduce food intake^23^. We found that high circulating GDF15 is associated with a substantially increased risk of hip fractures. Since most hip fractures are a result of a fall, also non-bone parameters may be important. Thus, it is interesting to note that high circulating GDF15 has been associated with a decline in gait speed and grip strength^24,25^. In addition, GDF15 is a negative regulator of muscle mass and suggested to be involved in sarcopenia^26-28^. Some, but not all, studies show that high circulating GDF15 is associated with reduced BMD or accelerated bone loss^28^ ^29^ ^30^ ^31^. At the cellular level, GDF15 has been shown to increase the number of multi-nucleated tartrate-resistant acid phosphatase (TRAP)-positive cells differentiated from human peripheral blood mononuclear cells and inhibit the alkaline phosphatase-activity and mineralization ability in human bone marrow-derived mesenchymal stem cells^32^. The effects of GDF15 are mediated via the transmembrane glial-derived neurotrophic factor (GDNF)-family receptor α-like (GFRAL), which recruits the transmembrane coreceptor tyrosine kinase RET (REarranged during Transfection) proto-oncogene^33,34^. Interestingly, high circulating RET associated with a decreased risk of hip fractures in the present study, leading us to speculate that circulating RET could be involved in inhibiting the actions of GDF15 on musculoskeletal health. High circulating WFDC2 associated with increased risk of hip fractures in the present study. This association may merely be caused by the strong positive correlation between circulating WFDC2 and circulating GDF15. WFDC2 is suggested to be an anti-proteinase that play a role in the immune system, but its possible role for bone health is unknown^35,36^. 

We observed that high circulating levels of CD14 associated with an increased risk of hip fractures. Similar results have previously been found in a subsample of the MrOS cohort^37^. In addition, higher soluble CD14 levels have previously been associated with increased risk of incident hip fractures in the CHS, when measured by ELISA^38^. Collectively, the evidence for CD14 as a reproducible biomarker of hip fracture risk is strong, with support from three studies using different methods (MS-technique, aptamer-based technique, and immunoassay) to analyze CD14 in the circulation. Mice lacking CD14 display increased bone strength, which is in line with the observational finding from the present study^39^. CD14 is part of the innate immune system and a receptor for lipopolysaccharide (LPS). It has been shown that CD14 has an important function in LPS-mediated bone resorption^40^. 

LPS also induces expression of the proinflammatory factor CXCL1^41^, and in the present study, high circulating CXCL12 associated with an increased risk of hip fractures. High plasma levels of CXCL12 have previously been shown to associate with reduced BMD^42,43^. One underlying mechanism may be that CXCL12 acts as a chemoattractant for circulating pre-osteoclasts and as a survival signal for mature osteoclasts^44^. CXCR4 is the major CXCL12 receptor, and pharmacological inhibition of this receptor prevents ovariectomy (ovx)-induced bone loss^45^. MMP12 and inter-α-trypsin-inhibitor (ITIH)3 are two other pro-inflammatory factors, and high levels of both of these two factors were associated with an increased risk of hip fractures in the present study^21,46,47^ ^48^. Thus, high levels of multiple circulating inflammatory-related factors (CD14, CXCL12, MMP12, ITIH3) were associated with increased risk of hip fractures in the present study.

High circulating levels of the bone morphogenetic protein (BMP) 4 antagonist CHRDL1 were associated with increased risk of hip fractures. In contrast, our MR analyses revealed that genetically determined increased CHRDL1 increased eBMD, an effect that was in the opposite direction to what was expected from the observational association with hip fractures. In a similar manner, directional differences between observational associations and MR findings have been observed for other circulating proteins^49-51^. For instance, opposite directions for observational associations and MR have been described for circulating NT-proBNP levels, which are higher in subjects with hypertension but are linked to natriuretic effects, which lower blood pressure^49^. Thus, opposite directions for causal and observational associations might be due to a feed-back mechanism in which a protein (NT-proBNP) is observationally directly associated with a trait (blood pressure) while its physiological function is to reduce the same trait (blood pressure). In addition, it is possible that circulating levels of a specific protein is mainly dependent on environmental factors with no/minor genetic influence, limiting the usefulness of MR analyses to determine the causality of this protein. Interestingly, it has been described that the heritability of circulating CHRDL1 is rather low, with 90% being environmentally determined^47^. Therefore, further mechanistic studies are required to determine how circulating CHRDL1 influences hip fracture risk. 

Lipocalin 2 (LCN2) is an osteoblast-derived circulating factor that reduces food intake. We observed that high circulating LCN2 associates with increased hip fracture risk^52,53^, findings supported by a previous study showing that circulating levels of LCN2 predicts hip fracture risk in elderly women^54^. A negative effect of LCN2 on bone health is supported by the fact that transgenic mice overexpressing LCN2 in osteoblasts have reduced bone mass^55^. 

High levels of circulating spondin 1 (SPON1), a cell adhesion protein, were associated with an increased risk of hip fractures. Our results are supported by studies showing that the *SPON1* locus is associated with total hip BMD^56^ and that *SPON1* deficient mice have increased bone mass, suggesting that SPON1 is a negative regulator of bone mass^57^. 

In addition to the above discussed proteins, we identified several proteins associated with increased risk of hip fractures: high circulating levels of the canonical Wnt/beta-catenin enhancer R-spondin-1 (RSPO1); vesicular integral-membrane protein VIP36 (LMAN2), related with cellular endoplasmic reticulum stress; secretogranin-1 (CHGB), a neuroendocrine secretory granule protein; complex of serine protease kallikrein 3 and protease inhibitor α-1-antichymotrypsin (KLK3/SERPINA3 complex), where SERPINA3 is an acute phase protein; and protein delta homolog 2 (DLK2), a member of the epidermal growth factor (EGF)-like repeat-containing family. Several other proteins were associated with decreased risk of hip fractures: high circulating levels of bifunctional heparan sulfate N-deacetylase/N-sulfotransferase 1 (NDST1), a member of the enzyme family catalyzing the first modification step in the biosynthesis of heparan sulfate; neuropeptide S (NPS), a secretory protein mainly expressed in the brain; leptin (LEP), a fat mass regulating protein; and glycolipid transfer protein domain containing 2 (GLTPD2*)*, a member of the GLTP superfamily which are sphingolipid transfer/binding proteins. The underlying mechanisms of these associations remains to be determined.

# References

1.	Gold, L., Ayers, D., Bertino, J., Bock, C., Bock, A., Brody, E.N., Carter, J., Dalby, A.B., Eaton, B.E., Fitzwater, T., et al. (2010). Aptamer-based multiplexed proteomic technology for biomarker discovery. PLoS One *5*, e15004. [https://doi.org/10.1371/journal.pone.0015004](https://doi.org/10.1371/journal.pone.0015004).

2.	Austin, T.R., McHugh, C.P., Brody, J.A., Bis, J.C., Sitlani, C.M., Bartz, T.M., Biggs, M.L., Bansal, N., Buzkova, P., Carr, S.A., et al. (2022). Proteomics and Population Biology in the Cardiovascular Health Study (CHS): design of a study with mentored access and active data sharing. Eur J Epidemiol *37*, 755-765. [https://doi.org/10.1007/s10654-022-00888-z](https://doi.org/10.1007/s10654-022-00888-z).

3.	Lollo, B., Steele, F., and Gold, L. (2014). Beyond antibodies: new affinity reagents to unlock the proteome. Proteomics *14*, 638-644. [https://doi.org/10.1002/pmic.201300187](https://doi.org/10.1002/pmic.201300187).

4.	Lindbohm, J.V., Mars, N., Walker, K.A., Singh-Manoux, A., Livingston, G., Brunner, E.J., Sipila, P.N., Saksela, K., Ferrie, J.E., Lovering, R.C., et al. (2022). Plasma proteins, cognitive decline, and 20-year risk of dementia in the Whitehall II and Atherosclerosis Risk in Communities studies. Alzheimers Dement *18*, 612-624. [https://doi.org/10.1002/alz.12419](https://doi.org/10.1002/alz.12419).

5.	Candia, J., Cheung, F., Kotliarov, Y., Fantoni, G., Sellers, B., Griesman, T., Huang, J., Stuccio, S., Zingone, A., Ryan, B.M., et al. (2017). Assessment of Variability in the SOMAscan Assay. Sci Rep *7*, 14248. [https://doi.org/10.1038/s41598-017-14755-5](https://doi.org/10.1038/s41598-017-14755-5).

6.	Schilbach, K., and Bidlingmaier, M. (2015). Growth hormone binding protein - physiological and analytical aspects. Best Pract Res Clin Endocrinol Metab *29*, 671-683. [https://doi.org/10.1016/j.beem.2015.06.004](https://doi.org/10.1016/j.beem.2015.06.004).

7.	de Vos, A.M., Ultsch, M., and Kossiakoff, A.A. (1992). Human growth hormone and extracellular domain of its receptor: crystal structure of the complex. Science *255*, 306-312. [https://doi.org/10.1126/science.1549776](https://doi.org/10.1126/science.1549776).

8.	Rosen, T., Wilhelmsen, L., Landin-Wilhelmsen, K., Lappas, G., and Bengtsson, B.A. (1997). Increased fracture frequency in adult patients with hypopituitarism and GH deficiency. Eur J Endocrinol *137*, 240-245. [https://doi.org/10.1530/eje.0.1370240](https://doi.org/10.1530/eje.0.1370240).

9.	Wuster, C., Abs, R., Bengtsson, B.A., Bennmarker, H., Feldt-Rasmussen, U., Hernberg-Stahl, E., Monson, J.P., Westberg, B., Wilton, P., Group, K.S., et al. (2001). The influence of growth hormone deficiency, growth hormone replacement therapy, and other aspects of hypopituitarism on fracture rate and bone mineral density. J Bone Miner Res *16*, 398-405. [https://doi.org/10.1359/jbmr.2001.16.2.398](https://doi.org/10.1359/jbmr.2001.16.2.398).

10.	Mo, D., Fleseriu, M., Qi, R., Jia, N., Child, C.J., Bouillon, R., and Hardin, D.S. (2015). Fracture risk in adult patients treated with growth hormone replacement therapy for growth hormone deficiency: a prospective observational cohort study. Lancet Diabetes Endocrinol *3*, 331-338. [https://doi.org/10.1016/S2213-8587(15)00098-4](https://doi.org/10.1016/S2213-8587(15)00098-4).

11.	Landin-Wilhelmsen, K., Nilsson, A., Bosaeus, I., and Bengtsson, B.A. (2003). Growth hormone increases bone mineral content in postmenopausal osteoporosis: a randomized placebo-controlled trial. J Bone Miner Res *18*, 393-405. [https://doi.org/10.1359/jbmr.2003.18.3.393](https://doi.org/10.1359/jbmr.2003.18.3.393).

12.	Kassem, M., Brixen, K., Mosekilde, L., Blum, W.F., and Flyvbjerg, A. (1998). Effects of growth hormone treatment on serum levels of insulin-like growth factors (IGFs) and IGF binding proteins 1-4 in postmenopausal women. Clin Endocrinol (Oxf) *49*, 747-756. [https://doi.org/10.1046/j.1365-2265.1998.00606.x](https://doi.org/10.1046/j.1365-2265.1998.00606.x).

13.	Jehle, P.M., Schulten, K., Schulz, W., Jehle, D.R., Stracke, S., Manfras, B., Boehm, B.O., Baylink, D.J., and Mohan, S. (2003). Serum levels of insulin-like growth factor (IGF)-I and IGF binding protein (IGFBP)-1 to -6 and their relationship to bone metabolism in osteoporosis patients. Eur J Intern Med *14*, 32-38. [https://doi.org/10.1016/s0953-6205(02)00183-8](https://doi.org/10.1016/s0953-6205(02)00183-8).

14.	Maramotti, S., Paci, M., Manzotti, G., Rapicetta, C., Gugnoni, M., Galeone, C., Cesario, A., and Lococo, F. (2016). Soluble Epidermal Growth Factor Receptors (sEGFRs) in Cancer: Biological Aspects and Clinical Relevance. Int J Mol Sci *17*. [https://doi.org/10.3390/ijms17040593](https://doi.org/10.3390/ijms17040593).

15.	Liu, G., Xie, Y., Su, J., Qin, H., Wu, H., Li, K., Yu, B., and Zhang, X. (2019). The role of EGFR signaling in age-related osteoporosis in mouse cortical bone. FASEB J *33*, 11137-11147. [https://doi.org/10.1096/fj.201900436RR](https://doi.org/10.1096/fj.201900436RR).

16.	Linder, M., Hecking, M., Glitzner, E., Zwerina, K., Holcmann, M., Bakiri, L., Ruocco, M.G., Tuckermann, J., Schett, G., Wagner, E.F., and Sibilia, M. (2018). EGFR controls bone development by negatively regulating mTOR-signaling during osteoblast differentiation. Cell Death Differ *25*, 1094-1106. [https://doi.org/10.1038/s41418-017-0054-7](https://doi.org/10.1038/s41418-017-0054-7).

17.	Camaj, P., Seeliger, H., Ischenko, I., Krebs, S., Blum, H., De Toni, E.N., Faktorova, D., Jauch, K.W., and Bruns, C.J. (2009). EFEMP1 binds the EGF receptor and activates MAPK and Akt pathways in pancreatic carcinoma cells. Biol Chem *390*, 1293-1302. [https://doi.org/10.1515/BC.2009.140](https://doi.org/10.1515/BC.2009.140).

18.	Kim, I.G., Kim, S.Y., Choi, S.I., Lee, J.H., Kim, K.C., and Cho, E.W. (2014). Fibulin-3-mediated inhibition of epithelial-to-mesenchymal transition and self-renewal of ALDH+ lung cancer stem cells through IGF1R signaling. Oncogene *33*, 3908-3917. [https://doi.org/10.1038/onc.2013.373](https://doi.org/10.1038/onc.2013.373).

19.	Sathyan, S., Ayers, E., Gao, T., Weiss, E.F., Milman, S., Verghese, J., and Barzilai, N. (2020). Plasma proteomic profile of age, health span, and all-cause mortality in older adults. Aging Cell *19*, e13250. [https://doi.org/10.1111/acel.13250](https://doi.org/10.1111/acel.13250).

20.	Cominetti, O., Nunez Galindo, A., Corthesy, J., Valsesia, A., Irincheeva, I., Kussmann, M., Saris, W.H.M., Astrup, A., McPherson, R., Harper, M.E., et al. (2018). Obesity shows preserved plasma proteome in large independent clinical cohorts. Sci Rep *8*, 16981. [https://doi.org/10.1038/s41598-018-35321-7](https://doi.org/10.1038/s41598-018-35321-7).

21.	Tanaka, T., Biancotto, A., Moaddel, R., Moore, A.Z., Gonzalez-Freire, M., Aon, M.A., Candia, J., Zhang, P., Cheung, F., Fantoni, G., et al. (2018). Plasma proteomic signature of age in healthy humans. Aging Cell *17*, e12799. [https://doi.org/10.1111/acel.12799](https://doi.org/10.1111/acel.12799).

22.	Hsu, Y.-H., Kwon, R., Kinyua, F., On Wong, A., Rai, J., Hulleman, J., Watson, C., Okoro, P., and et.al (2022). EFEMP1 regulates bone structure and affects fracture risk: a discovery from HR-pQCT bone microarchitecture GWAS screening confirmed by CRISPR knockout zebrafishes and mice: The bone microarchitecture international consortium (BoMIC). American Society for Bone and Mineral Research.

23.	Tsai, V.W.W., Husaini, Y., Sainsbury, A., Brown, D.A., and Breit, S.N. (2018). The MIC-1/GDF15-GFRAL Pathway in Energy Homeostasis: Implications for Obesity, Cachexia, and Other Associated Diseases. Cell Metab *28*, 353-368. [https://doi.org/10.1016/j.cmet.2018.07.018](https://doi.org/10.1016/j.cmet.2018.07.018).

24.	Liu, X., Pan, S., Xanthakis, V., Vasan, R.S., Psaty, B.M., Austin, T.R., Newman, A.B., Sanders, J.L., Wu, C., Tracy, R.P., et al. (2022). Plasma proteomic signature of decline in gait speed and grip strength. Aging Cell, e13736. [https://doi.org/10.1111/acel.13736](https://doi.org/10.1111/acel.13736).

25.	Grisso, J.A., Kelsey, J.L., Strom, B.L., Chiu, G.Y., Maislin, G., O'Brien, L.A., Hoffman, S., and Kaplan, F. (1991). Risk factors for falls as a cause of hip fracture in women. The Northeast Hip Fracture Study Group. N Engl J Med *324*, 1326-1331. [https://doi.org/10.1056/NEJM199105093241905](https://doi.org/10.1056/NEJM199105093241905).

26.	Kalinkovich, A., and Livshits, G. (2015). Sarcopenia--The search for emerging biomarkers. Ageing Res Rev *22*, 58-71. [https://doi.org/10.1016/j.arr.2015.05.001](https://doi.org/10.1016/j.arr.2015.05.001).

27.	Alcazar, J., Frandsen, U., Prokhorova, T., Kamper, R.S., Haddock, B., Aagaard, P., and Suetta, C. (2021). Changes in systemic GDF15 across the adult lifespan and their impact on maximal muscle power: the Copenhagen Sarcopenia Study. J Cachexia Sarcopenia Muscle *12*, 1418-1427. [https://doi.org/10.1002/jcsm.12823](https://doi.org/10.1002/jcsm.12823).

28.	Lee, S.H., Lee, J.Y., Lim, K.H., Lee, Y.S., and Koh, J.M. (2022). Associations Between Plasma Growth and Differentiation Factor-15 with Aging Phenotypes in Muscle, Adipose Tissue, and Bone. Calcif Tissue Int *110*, 236-243. [https://doi.org/10.1007/s00223-021-00912-6](https://doi.org/10.1007/s00223-021-00912-6).

29.	Li, W., Li, C., Zhou, X., Jiang, T., Guo, L., and Liu, H. (2020). Relationship between GDF15 level and bone metabolism in postmenopausal Chinese women. Gynecol Endocrinol *36*, 714-717. [https://doi.org/10.1080/09513590.2020.1764929](https://doi.org/10.1080/09513590.2020.1764929).

30.	Hong, S.W., and Kang, J.H. (2022). Growth differentiation factor-15 as a modulator of bone and muscle metabolism. Front Endocrinol (Lausanne) *13*, 948176. [https://doi.org/10.3389/fendo.2022.948176](https://doi.org/10.3389/fendo.2022.948176).

31.	Osawa, Y., Tanaka, T., Semba, R.D., Fantoni, G., Moaddel, R., Candia, J., Simonsick, E.M., Bandinelli, S., and Ferrucci, L. (2022). Plasma growth and differentiation factor 15 predict longitudinal changes in bone parameters in women, but not in men. J Gerontol A Biol Sci Med Sci. [https://doi.org/10.1093/gerona/glac079](https://doi.org/10.1093/gerona/glac079).

32.	Westhrin, M., Moen, S.H., Holien, T., Mylin, A.K., Heickendorff, L., Olsen, O.E., Sundan, A., Turesson, I., Gimsing, P., Waage, A., and Standal, T. (2015). Growth differentiation factor 15 (GDF15) promotes osteoclast differentiation and inhibits osteoblast differentiation and high serum GDF15 levels are associated with multiple myeloma bone disease. Haematologica *100*, e511-514. [https://doi.org/10.3324/haematol.2015.124511](https://doi.org/10.3324/haematol.2015.124511).

33.	Emmerson, P.J., Wang, F., Du, Y., Liu, Q., Pickard, R.T., Gonciarz, M.D., Coskun, T., Hamang, M.J., Sindelar, D.K., Ballman, K.K., et al. (2017). The metabolic effects of GDF15 are mediated by the orphan receptor GFRAL. Nat Med *23*, 1215-1219. [https://doi.org/10.1038/nm.4393](https://doi.org/10.1038/nm.4393).

34.	Yang, L., Chang, C.C., Sun, Z., Madsen, D., Zhu, H., Padkjaer, S.B., Wu, X., Huang, T., Hultman, K., Paulsen, S.J., et al. (2017). GFRAL is the receptor for GDF15 and is required for the anti-obesity effects of the ligand. Nat Med *23*, 1158-1166. [https://doi.org/10.1038/nm.4394](https://doi.org/10.1038/nm.4394).

35.	Zhan, Y., Chen, J., Wu, J., Gu, Y., Huang, Q., Deng, Z., Chen, S., Wu, X., Lv, Y., Zeng, Z., and Xie, J. (2022). Human epididymis protein 4 aggravates airway inflammation and remodeling in chronic obstructive pulmonary disease. Respir Res *23*, 120. [https://doi.org/10.1186/s12931-022-02040-7](https://doi.org/10.1186/s12931-022-02040-7).

36.	Nagy, B., Jr., Nagy, B., Fila, L., Clarke, L.A., Gonczy, F., Bede, O., Nagy, D., Ujhelyi, R., Szabo, A., Anghelyi, A., et al. (2016). Human Epididymis Protein 4: A Novel Serum Inflammatory Biomarker in Cystic Fibrosis. Chest *150*, 661-672. [https://doi.org/10.1016/j.chest.2016.04.006](https://doi.org/10.1016/j.chest.2016.04.006).

37.	Nielson, C.M., Wiedrick, J., Shen, J., Jacobs, J., Baker, E.S., Baraff, A., Piehowski, P., Lee, C.G., Baratt, A., Petyuk, V., et al. (2017). Identification of Hip BMD Loss and Fracture Risk Markers Through Population-Based Serum Proteomics. J Bone Miner Res *32*, 1559-1567. [https://doi.org/10.1002/jbmr.3125](https://doi.org/10.1002/jbmr.3125).

38.	Bethel, M., Buzkova, P., Fink, H.A., Robbins, J.A., Cauley, J.A., Lee, J., Barzilay, J.I., Jalal, D.I., and Carbone, L.D. (2016). Soluble CD14 and fracture risk. Osteoporos Int *27*, 1755-1763. [https://doi.org/10.1007/s00198-015-3439-9](https://doi.org/10.1007/s00198-015-3439-9).

39.	Johnson, G.B., Riggs, B.L., and Platt, J.L. (2004). A genetic basis for the "Adonis" phenotype of low adiposity and strong bones. FASEB J *18*, 1282-1284. [https://doi.org/10.1096/fj.04-1572fje](https://doi.org/10.1096/fj.04-1572fje).

40.	Amano, S., Kawakami, K., Iwahashi, H., Kitano, S., and Hanazawa, S. (1997). Functional role of endogenous CD14 in lipopolysaccharide-stimulated bone resorption. J Cell Physiol *173*, 301-309. [https://doi.org/10.1002/(SICI)1097-4652(199712)173:3](https://doi.org/10.1002/(SICI)1097-4652(199712)173:3)<301::AID-JCP1>3.0.CO;2-R.

41.	Shima, K., Kimura, K., Ishida, M., Kishikawa, A., Ogawa, S., Qi, J., Shen, W.R., Ohori, F., Noguchi, T., Marahleh, A., and Kitaura, H. (2018). C-X-C Motif Chemokine 12 Enhances Lipopolysaccharide-Induced Osteoclastogenesis and Bone Resorption In Vivo. Calcif Tissue Int *103*, 431-442. [https://doi.org/10.1007/s00223-018-0435-z](https://doi.org/10.1007/s00223-018-0435-z).

42.	Carbone, L.D., Buzkova, P., Fink, H.A., Robbins, J.A., Bethel, M., Hamrick, M.W., and Hill, W.D. (2017). Association of Plasma SDF-1 with Bone Mineral Density, Body Composition, and Hip Fractures in Older Adults: The Cardiovascular Health Study. Calcif Tissue Int *100*, 599-608. [https://doi.org/10.1007/s00223-017-0245-8](https://doi.org/10.1007/s00223-017-0245-8).

43.	Yang, X.W., Huang, H.X., Wang, F., Zhou, Q.L., Huang, Y.Q., and Qin, R.Z. (2020). Elevated plasma CXCL12/SDF-1 levels are linked with disease severity of postmenopausal osteoporosis. Innate Immun *26*, 222-230. [https://doi.org/10.1177/1753425919883365](https://doi.org/10.1177/1753425919883365).

44.	Wright, L.M., Maloney, W., Yu, X., Kindle, L., Collin-Osdoby, P., and Osdoby, P. (2005). Stromal cell-derived factor-1 binding to its chemokine receptor CXCR4 on precursor cells promotes the chemotactic recruitment, development and survival of human osteoclasts. Bone *36*, 840-853. [https://doi.org/10.1016/j.bone.2005.01.021](https://doi.org/10.1016/j.bone.2005.01.021).

45.	Im, J.Y., Min, W.K., Park, M.H., Kim, N., Lee, J.K., Jin, H.K., Choi, J.Y., Kim, S.Y., and Bae, J.S. (2014). AMD3100 improves ovariectomy-induced osteoporosis in mice by facilitating mobilization of hematopoietic stem/progenitor cells. BMB Rep *47*, 439-444. [https://doi.org/10.5483/bmbrep.2014.47.8.159](https://doi.org/10.5483/bmbrep.2014.47.8.159).

46.	Lehallier, B., Gate, D., Schaum, N., Nanasi, T., Lee, S.E., Yousef, H., Moran Losada, P., Berdnik, D., Keller, A., Verghese, J., et al. (2019). Undulating changes in human plasma proteome profiles across the lifespan. Nat Med *25*, 1843-1850. [https://doi.org/10.1038/s41591-019-0673-2](https://doi.org/10.1038/s41591-019-0673-2).

47.	Menni, C., Kiddle, S.J., Mangino, M., Vinuela, A., Psatha, M., Steves, C., Sattlecker, M., Buil, A., Newhouse, S., Nelson, S., et al. (2015). Circulating Proteomic Signatures of Chronological Age. J Gerontol A Biol Sci Med Sci *70*, 809-816. [https://doi.org/10.1093/gerona/glu121](https://doi.org/10.1093/gerona/glu121).

48.	Jiang, X., Bai, X.Y., Li, B., Li, Y., Xia, K., Wang, M., Li, S., and Wu, H. (2019). Plasma Inter-Alpha-Trypsin Inhibitor Heavy Chains H3 and H4 Serve as Novel Diagnostic Biomarkers in Human Colorectal Cancer. Dis Markers *2019*, 5069614. [https://doi.org/10.1155/2019/5069614](https://doi.org/10.1155/2019/5069614).

49.	Theriault, S., Sjaarda, J., Chong, M., Hess, S., Gerstein, H., and Pare, G. (2020). Identification of Circulating Proteins Associated With Blood Pressure Using Mendelian Randomization. Circ Genom Precis Med *13*, e002605. [https://doi.org/10.1161/CIRCGEN.119.002605](https://doi.org/10.1161/CIRCGEN.119.002605).

50.	Gudmundsdottir, V., Zaghlool, S.B., Emilsson, V., Aspelund, T., Ilkov, M., Gudmundsson, E.F., Jonsson, S.M., Zilhao, N.R., Lamb, J.R., Suhre, K., et al. (2020). Circulating Protein Signatures and Causal Candidates for Type 2 Diabetes. Diabetes *69*, 1843-1853. [https://doi.org/10.2337/db19-1070](https://doi.org/10.2337/db19-1070).

51.	Matias-Garcia, P.R., Wilson, R., Guo, Q., Zaghlool, S.B., Eales, J.M., Xu, X., Charchar, F.J., Dormer, J., Maalmi, H., Schlosser, P., et al. (2021). Plasma Proteomics of Renal Function: A Transethnic Meta-Analysis and Mendelian Randomization Study. J Am Soc Nephrol *32*, 1747-1763. [https://doi.org/10.1681/ASN.2020071070](https://doi.org/10.1681/ASN.2020071070).

52.	Dekens, D.W., Eisel, U.L.M., Gouweleeuw, L., Schoemaker, R.G., De Deyn, P.P., and Naude, P.J.W. (2021). Lipocalin 2 as a link between ageing, risk factor conditions and age-related brain diseases. Ageing Res Rev *70*, 101414. [https://doi.org/10.1016/j.arr.2021.101414](https://doi.org/10.1016/j.arr.2021.101414).

53.	Mosialou, I., Shikhel, S., Liu, J.M., Maurizi, A., Luo, N., He, Z., Huang, Y., Zong, H., Friedman, R.A., Barasch, J., et al. (2017). MC4R-dependent suppression of appetite by bone-derived lipocalin 2. Nature *543*, 385-390. [https://doi.org/10.1038/nature21697](https://doi.org/10.1038/nature21697).

54.	Lim, W.H., Wong, G., Lim, E.M., Byrnes, E., Zhu, K., Devine, A., Pavlos, N.J., Prince, R.L., and Lewis, J.R. (2015). Circulating Lipocalin 2 Levels Predict Fracture-Related Hospitalizations in Elderly Women: A Prospective Cohort Study. J Bone Miner Res *30*, 2078-2085. [https://doi.org/10.1002/jbmr.2546](https://doi.org/10.1002/jbmr.2546).

55.	Costa, D., Lazzarini, E., Canciani, B., Giuliani, A., Spano, R., Marozzi, K., Manescu, A., Cancedda, R., and Tavella, S. (2013). Altered bone development and turnover in transgenic mice over-expressing lipocalin-2 in bone. J Cell Physiol *228*, 2210-2221. [https://doi.org/10.1002/jcp.24391](https://doi.org/10.1002/jcp.24391).

56.	Gregson, C.L., Newell, F., Leo, P.J., Clark, G.R., Paternoster, L., Marshall, M., Forgetta, V., Morris, J.A., Ge, B., Bao, X., et al. (2018). Genome-wide association study of extreme high bone mass: Contribution of common genetic variation to extreme BMD phenotypes and potential novel BMD-associated genes. Bone *114*, 62-71. [https://doi.org/10.1016/j.bone.2018.06.001](https://doi.org/10.1016/j.bone.2018.06.001).

57.	Palmer, G.D., Attur, M.G., Yang, Q., Liu, J., Moon, P., Beier, F., and Abramson, S.B. (2014). F-spondin deficient mice have a high bone mass phenotype. PLoS One *9*, e98388. [https://doi.org/10.1371/journal.pone.0098388](https://doi.org/10.1371/journal.pone.0098388).
